# *Schistosoma haematobium* infections among schoolchildren in central Sudan one year after treatment with praziquantel

**DOI:** 10.1186/1756-3305-5-108

**Published:** 2012-06-07

**Authors:** Abedaziz M Ahmed, Hana Abbas, Fathi A Mansour, Gasim I Gasim, Ishag Adam

**Affiliations:** 1 Schistosomiasis Research Laboratory, Faculty of Science, University of Khartoum, Khartoum, Sudan; 2 Department of Epidemiology, Tropical Medicine Research Institute, Khartoum, Sudan; 3 Faculty of Medicine, Qassim University, Qassim, Kingdom of Saudi Arabia; 4 Faculty of Medicine, University of Khartoum, P.O. Box 102, Khartoum, Sudan

## Abstract

**Background:**

Chemotherapy with praziquantel (PZQ) is the mainstay of schistosomiasis control. However, there are recent concerns about tolerance or resistance to PZQ, so that monitoring its efficacy in different settings is required.

**Methods:**

A longitudinal study was conducted to evaluate the impact of PZQ for the treatment of *Schistosoma haematobium* infection among schoolchildren at Al Salamania, Central Sudan. Parasitological examinations for *S. haematobium* were performed in a cohort of schoolchildren (6–15 years of age) before and 1 year after treatment with a single dose of PZQ 40 mg/kg.

**Results:**

Out of 562 (309 boys and 253 girls) schoolchildren recruited from three elementary schools, 420 completed one longitudinal dataset that comprised of data from two time points; baseline, and follow-up 1 year after treatment with a single dose of PZQ 40 mg/kg for *S. haematobium* infection. A single dose of PZQ significantly reduced the prevalence of *S. haematobium* infection by 83.3% (from 51.4% to 8.6%) and the geometric mean intensity of infection of positive individuals by 17.0% (from 87.7 to 72.8 eggs/10 ml of urine) 1 year after treatment. While there was no significant difference in the reduction of the prevalence of *S. haematobium* infection between the gender or age groups, there was a significantly higher reduction of intensity of *S. haematobium* infection among girls in comparison with boys.

**Conclusion:**

There was a significant reduction of *S. haematobium* infection 1 year after PZQ treatment in this setting.

## Background

Schistosomiasis is endemic in 74 countries [1], with a bulk of the global cases (90%) residing in sub-Saharan Africa [1,2]. In 2007, the World Health Organization estimated 235 million cases of schistosomiasis worldwide, with 732 million people at risk of infection in known transmission areas [3]. In 2000, it was estimated that 70 million people had hematuria, 32 million had dysuria associated with *Schistosoma haematobium*, 18 million had major bladder wall pathology, 10 million people had *S. haematobium* related renal failure; and schistosomiasis related bladder cancer, resulting in an estimated mortality of 150 000 people per year in sub- Saharan Africa [4]. In Sudan the risk for *S. haematobium* is widespread in the different regions [5-7] and school age children were at a higher risk of *S. haematobium* infection than the other age groups [6]. Due to many factors such as higher rates of water activities, anatomical vasculature supplying genitourinary structures and immunological factors, school-aged children are the group at highest risk of contracting *S. haematobium* infection [8-10].

Praziquantel (PZQ) is the mainstay of the current strategy against schistosomiasis morbidity control [2,11]. World Health Organization (WHO) guidelines recommend that in communities with schistosomiasis, and a prevalence of 10% up to 50%, school-aged children and high-risk groups of adults should be treated with PZQ once every two years. In communities where prevalence is 50% and above, the same groups should be treated once a year [12]. Therefore mass therapy with PZQ has been employed in many national control programmes for schistosomiasis across sub-Saharan Africa, including Sudan. Schools were the target for this policy and treatment because of increased benefits of reducing infection burdens in children compared to adults and the ease of providing treatment [13,14]. The assessment of using annual PZQ treatment for schoolchildren is highly needed for both caregivers and health planners. The present longitudinal study aimed to investigate the prevalence and intensity of *S. haematobium* infection 1 year after treatment with PZQ among schoolchildren at Al Salamania in central Sudan so as to add to the body of research on the epidemiology and effects of schistosomiasis infections among schoolchildren in Sudan [7,15,16].

## Methods

**Figure 1:**
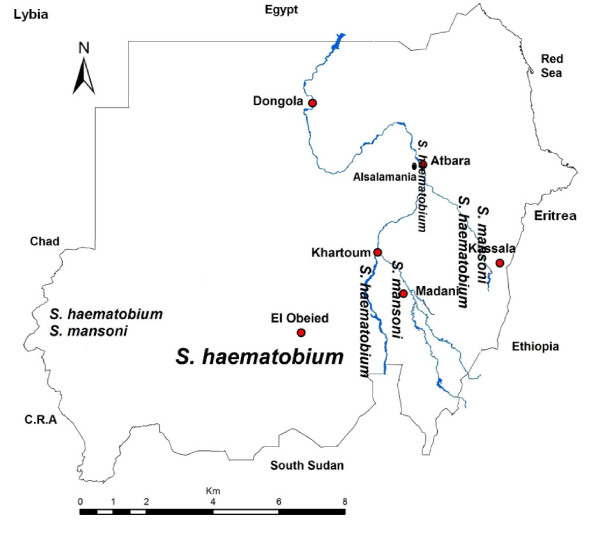
**Map of Sudan with study area and distribution of *S. haematobium* and *S. mansoni* based on the species name in the location**.

The details of the method have been mentioned in the previous work on *Schistosoma mansoni* infection [16]. In summary, this was a continuation of the collaborative projects between the University of Khartoum and the Ministry of Health in Sudan, and it was part of a pilot program for schistosomiasis (both mansoni and haematobium) control that aimed at treating schoolchildren for schistosomal infections. A longitudinal study was conducted at Al Salamania, (Figure 1) which is an agricultural area in central Sudan, during the period October 2008 to November 2009. The study was conducted in three primary schools (for boys and girls in levels from level 1 up to level 8, age between 1–16 years). All the children in these three schools were included in this study. Those students who received PZQ in the past 6 months were excluded from this study. After obtaining permission from the Education and Health Vice Chancellor and village head (sheikh) of this area, the study procedure was explained to the school authorities and parents/guardians of children. A longitudinal dataset that comprises data from two time points; baseline, and follow-up after 1 year was carried-out to determine the prevalence and intensity of urinary schistosomiasis among these schoolchildren. Students were provided with labeled 500 ml specimen containers and asked to provide a urine sample between 10.00 and 13.00/h. From each suspended sample, 10 ml were filtered using a 25-mm diameter filter holder and a Nucleopore® filter with a 12-μm pore size as previously described [17]. The filters were placed on a glass slide and examined quantitatively for *S. haematobium* eggs. Intensity of infection was expressed as eggs/10 ml of urine. Infection intensities were classified into two categories: (1) light infections (<50 eggs/10 ml of urine) and (2) heavy infections (≥50 eggs/10 ml of urine). For reducing the impact of zero egg count and normalization of the data the geometric mean of egg output was calculated in infected children only.

In the first survey, stool samples were examined for *S. mansoni* eggs and other helminths using the modified Kato technique for a single stool sample.

### Treatment

Treatment was given not only to the children in the three schools that were involved in the surveys, but to the entire children in all elementary schools in the whole district (18 schools). Treatment was carried out by health teams of trained nurses and medical officers as part of the treatment campaign. The study team assisted this health team with treatment and also recorded and observed the treatment of the study schoolchildren. All schoolchildren were treated for schistosome infection with a single dose of 40 mg/kg PZQ, without considering the infection status, 600 mg PZQ tablets, which can be subdivided into four segments of 150 mg were used. The health team provided a dosing sheet that showed the correct dosage for different body weights. Children infected with other helminthes received albendazole orally.

### Data analysis

Data were entered into the computer using SPSS for windows version 16.0 (SPSS Inc., Chicago, Illinois, USA) and double checked before analysis. The chi-square test was used to compare the differences in the prevalence (proportions) of infection. Student t-test was used to compare differences in the intensity of infection (eggs/10 ml of urine). A value of P < 0.05 was regarded as significant. Reduction in the prevalence and intensity (egg count in eggs/10 ml of urine) was calculated using the formulae below [18].

(1) $$\begin{array}{l} \begin{array}{cc} \text{Prevalence reduction }= & \begin{array}{c} \left(\%\text{ prevalence before treatment}-\%\text{ prevalence 1 year after treatment}\right)\\ /\left(\%\text{ prevalence before treatment}\right)\times 100\%. \end{array} \end{array}\\ \begin{array}{cc} \text{Reduction in the intensity }= & \begin{array}{c} \left(\text{meaneggs}/10\text{ ml of urinebefore treatment}-\text{meaneggs}/10\text{ ml of urine1 year after treatment}\right)\\ /(\text{meaneggs}/10\text{ ml of urinebefore treatment})\times 100\%\text{.} \end{array} \end{array} \end{array}$$

### Ethics

The study received ethical clearance from the Research Board of Communicable Disease, Ministry of Health, Sudan. The trial was registered under ClinicalTrials.gov NCT01558336

## Results

Of 562 (309 boys and 253 girls) schoolchildren enrolled at baseline from the three schools, 420 were successfully traced and re-examined at baseline and followed up with two survey results for longitudinal parasitological data on *S. haematobium*. There was no significant difference in the data of children who dropped-out or missed the follow-up survey to those included in both surveys (data not shown). The mean age of these 562 children was 9.7 years (range, 6–15 years). Because few cases of *S. mansoni* (2.3%), *Hymenolepis nana* (1.5%), hookworm (1.1%), *Trichuris trichiura* (1.1%), and *Ascaris lumbricoides* (0.01%) were detected in the first survey, stool examination was not conducted in the second survey.

Table 1 summarizes the parasitological results of *S. haematobium* infection in the children from three schools examined at baseline and 1 year after treatment. The prevalence of *S. haematobium* infection ranged from 38.5 to 59.2 1% with an overall prevalence of 51.4%, egg counts ranged from 71.7 to 104.4 eggs/10 ml of urine with a mean of 87.7 eggs/10 ml of urine among all children.

**Table 1:** The prevalence and intensity of *S. haematobium* infections in children from three schools in Al Salamania in Central Sudan

**Base line**				**One year post-treatment**		
**School**	**Children Examined**	**Prevalence**	**Intensity**	**Children Examined**	**Prevalence**	**Intensity**
	***n*(%)**	**%(95% CI)**	**(95% CI)**	***n*(%)**	**% (95% CI)**	**(95% CI)**
AbHaraz	157 (27.9)	59.2	86.9	153 (36.4)	13.1	73.3
		51.4─66.7	86.0─87.8		8.4─19.1	71.6─74.5
Karni	223 (39.7)	56.5	104.4	147 (35.0)	9.5	87.7
		50.0 ─62.0	103.4─105.4		5.5─15.1	84.7─90.7
El Dhiaga	182 (32.4)	38.5	71.7	(28.6)20	1.7	57.5
		31.6─45.7	71.0─72.5	1	0.3─5.3	21.5─93.4
Total	562 (100)	51.4	87.7	420 (100)	8.6	72.8
		47.3─55.5	87.3─88.1		6.2─11.5	71.5─74.1
P*		0.001	< 0.001		0.042	< 0.001

*P values indicate the difference between the schools.

While there was no significant difference in prevalence of infection between boys and girls (P = 0.228), boys had significantly higher intensity of *S. haematobium* infection than girls (P < 0.001, Table 2). Children of < 10 years of age had a significantly higher rate of prevalence (P = 0.001) and intensity of *S. haematobium* infection (P < 0 .001) than those children ≥ 10 years of age, (Table 3)

**Table 2:** The prevalence, intensity and reduction of *S. haematobium* infections in children from three schools in Al Salamania in Central Sudan at baseline and 1 year post treatment, relative to gender

**Variable**	**Total**	**Boys**	**Girls**	**P**
**Base line**				
Children examined, *n* (%)	562 (100)	309 (55.0)	253 (45.0)	
Prevalence,% (95% CI)	51.4(47.3─55.5)	53.7(48.1─59.2)	48.6(42.5─45.8)	0.228
Intensity, (95% CI)	87.7(87.2─88.1)	101.4(100.6─102.1)	74.0 (72.2─74.8)	<0.001
**One year post treatment**				
Children examined, *n* (%)	420 (100)	237(56.4)	183 (43.6)	
Prevalence,% (95% CI)	8.6(6.2─11.5)	8.9 (5.7─13.0)	8.2 (4.8─12.8)	0.809
Intensity, (95% CI)	72.8(71.5─74.1)	99.5(97.7─101.3)	46.2(44.3─48.1)	<0.001
Reduction in the prevalence,% (95% CI)	83.3 (73.0─89.7)	83.4(73.4─89.7)	83.1(73.5─90.3)	0.967
Reduction in the intensity, (95% CI)	17.0 (10.8─24.0)	1.9(0.6─3.5)	37.6(28.0─47.1)	0.001

**Table 3:** The prevalence, intensity and reduction of *S. haematobium* infections in children from three schools in Al Salamania in Central Sudan at baseline and 1 year post treatment, relative to age

**Variable**	**Total**	**Age < 10 years**	**Age ≥ 10 years**	**P**
**Base line**				
Children examined, *n* (%)	562 (100)	275 (49.0)	287 (51.0)	
Prevalence,% (95% CI)	51.4 (47.3─55.5)	58.2 (52.2─64.0)	44.9 (39.3─50.7)	0.001
Intensity, (95% CI)	87.7(87.2─88.1)	92.5 (91.8─93.1)	82.8 (82.1─83.4)	<0.001
**One year post treatment**				
Children examined, *n* (%)	420 (100)	205 (49.0)	215 (51.0)	
Prevalence,% (95% CI)	8.6 (6.2─11.5)	9.8 (6.2─4.4)	7.4(4.4─11.6)	0.397
Intensity, (95% CI)	72.8 (71.5─74.1)	74.4 (72.6─76.2)	71.2 (69.3─73.1)	0.014
Reduction in the prevalence,% (95% CI)	83.3 (73.0─89.7)	83.3(74.3─90.0)	83.5(73.4-90.6)	0.096
Reduction in the intensity, (95% CI)	17.0 (10.8─24.0)	19.6(13.7─27.8)	14.0 (8.6─21.1)	0.327

A single dose of PZQ significantly reduced the prevalence of *S. haematobium* infection by 83.3% (from 51.4% to 8.6%) and the intensity of infection by 17.0% (from 87.7 to 72.8 eggs/10 ml of urine) 1 year after treatment. While there was no significant difference in the reduction of the prevalence of *S. haematobium* infection between genders (P = 0.967) or age groups (P = 0.096), there was a significantly higher reduction of intensity of *S. haematobium* infection among girls in comparison with boys (P = 0.001, Table 2 and Table 3). There was no significant difference in the reduction of intensity among different age groups. Interestingly, the reduction in prevalence was not significantly different in children with heavy or light infections Table 4.

**Table 4:** Reduction in the prevalence of *S. haematobium* infections in children from three schools in Al Salamania in Central Sudan, relative to the level of infections

**Variable**	**Total**	**Light infection**	**Heavy infection**	**Negative**
**Base line**				
Children examined, *n* (%)	562 (100)	137(24.4)	152 (27.0)	273 (48.6)
Prevalence,% (95% CI)	51.4(47.3─55.5)	24.4 (21.0─28.0)	27.0 (23.5─30.8)	─
**One year post treatment**				
Children examined, *n* (%)	420 (100)	17(4.0)	19(4.5)	384 (91.4)
Prevalence,% (95% CI)	8.6(6.2─11.5)	4.0 ( 2.4─6.3)	4.5 (2.8─6.8)	─
Reduction in the prevalence,% (95% CI)	83.3 (73.0─89.7)	82.0 ( 63.1─93.7)	83.3 (70.8─91.4)	─

## Discussion

The main findings of the current study are; high prevalence and intensity of *S. haematobium* among these children, especially boys and among those less than ten years of age, a significant reduction in prevalence [83.3% (from 51.4% to 8.6%)] and intensity of *S. haematobium* infection [17.0% (from 87.7 to 72.8 eggs/10 ml of urine)] 1 year after PZQ treatment. While there was no significant difference in reduction of prevalence of *S. haematobium* infection between gender, age groups or level of infection, there was a significantly higher reduction of intensity of *S. haematobium* infection among girls in comparison to boys. The prevalence and intensity of infection of *S. haematobium* in this setting indicates that according to WHO criteria, regular treatment of schoolchildren in the area is indeed necessary [12].

The different age patterns for prevalence of infection of *S. haematobium* and differences in infection intensity observed in boys might indicate different water contact patterns.

Recently, Touré *et al*., [19] observed that a single round of PZQ treatment significantly reduced prevalence of *S. haematobium* infection by 87% (from 59.6% to 7.7%) and intensity of infection by 92.8% (from 94.2 to 6.8 eggs/10 ml of urine) two years post-treatment. Moreover, the proportion of school-age children with heavy *S. haematobium* infection decreased significantly (from 25% before treatment to around 2–3%) two years post-treatment. In Mozambique, two cohorts of *S. haematobium* infected schoolchildren were followed up two months after PZQ treatment. The prevalence of infection decreased from 54.2% and 51.7% in high and low transmission seasons to 30.3% and 1.8%, respectively. The intensity of infection decreased from 23.3 eggs/10 ml of urine at baseline to 15.6 eggs/10 ml of urine in children treated during high transmission season, and from 23.5 eggs/10 ml urine to 7.3 eggs/10 ml of urine children treated during low transmission season [20]. In Niger, three years after a single PZQ treatment, prevalence and intensity of *S. haematobium* infection remained significantly lower than at baseline [21]. It is worth mentioning that different measures of intensity (arithmetic mean of all positive and negative individuals) were used in the other setting [22]; therefore the results are not directly comparable with this study. Previous reports from Khartoum, Sudan showed that PZQ treatment of schoolchildren infected with *S. haematobium* and/or *S. mansoni* was highly effective in terms of cure rate (58%) and reduction (98%) in egg count six weeks after the treatment [5].

We have recently shown that a single dose of PZQ reduced the overall prevalence of *S. mansoni* infection by 36.7% (from 59.1 to 37.4%) and the intensity of infection by 41.1% (from 116.7 to 68.7 eggs per gram of stool) 1 year after treatment. The reduction in prevalence was significantly higher among the group of children with heavy infections (by 76.1%, from 6.7 to 1.6%) and among girls (by 54.1%, 42.3 to 19.4%) at 1 year after treatment [16]. Because infections with *S. haematobium* are more prevalent and generally more pathogenic in sub-Saharan Africa [4], perhaps more PZQ should be used against *S. haematobium* rather than *S. mansoni*, [4]. Evidence suggests that *S. haematobium* is more sensitive to PZQ than *S. mansoni*[23]. Yet, there are some reports of *S. haematobium* infections that failed to respond to PZQ [24,25].

In the current study, there was no significant difference in reduction of prevalence of *S. haematobium* infection between the levels of infection. Recent reports showed that children lightly infected with *S. haematobium* had a better cure rate than heavily infected children [20]. Previously, Utzinger *et al*. and Raso *et al*. [26,27] found an inverse relationship between cure rate and intensity of infection in intestinal schistosomiasis. A recent systematic review and meta-analysis showed that praziquantel is an effective agent in schistosomiasis treatment, and multiple doses might improve its efficacy [28].

In schistosomiasis, infection intensity is a better indicator of morbidity than prevalence as it reflects the number of worms infecting the individual and it is also a more reliable marker of treatment success defined as the removal of egg-laying worms, which is usually the case in treatment programs and larger epidemiological studies [18,29,30]. One of the limitations of the current study is the use of single egg counts. A single egg count is a less reliable tool for estimating prevalence and infection status of schistosomiasis. The examination of two or more specimens per child would have led to even higher estimates of total prevalence and intensity. The other limitation of the current work is lack of information on seasonality of the transmission of *S. haematobium* infection in the area, which might have influenced the response to PZQ treatment. Recent reports showed that adults and preschool children were at risk of schistosomiasis and can contribute to the transmission [31,32]; therefore schistosomiasis control interventions should also target these groups in addition to school children in endemic areas.

## Conclusion

There was a significant reduction of *S. haematobium* infection 1 year after PZQ treatment in this setting.

## Competing interests

The authors declare that they have no competing interests.

## Authors’ contributions

AAA and IA designed the study. HA, FAM and GIG carried out the study and participated in the statistical analysis and procedures. AAA and IA coordinated and participated in the design of the study, statistical analysis and the drafting of the manuscript. All the authors read and approved the final version.
